# *Rhus glabra* L. and *Pistacia vera* L. Leaf Extracts as Rich Sources of Tannins with Antioxidant, Anticancer and Antimicrobial Activities

**DOI:** 10.3390/molecules31203739

**Published:** 2026-10-18

**Authors:** Elżbieta Okła, Ewa Olchowik-Grabarek, Paulina Rycharska, Rustamjon Makhmudov, Rakhmatilla Rakhimov, Nodira Abdulladjanova, Janusz Błasiak, Maria Zamaraeva, Maksim Ionov

**Affiliations:** 1 Department of General Biophysics, Faculty of Biology and Environmental Protection, University of Lodz, Pomorska 141/143, 90-236 Lodz, Poland; elzbieta.okla@biol.uni.lodz.pl (E.O.); paulina.rycharska@edu.uni.lodz.pl (P.R.); maksim.ionov@biol.uni.lodz.pl (M.I.); 2 Department of Microbiology and Biotechnology, Faculty of Biology, University of Bialystok, 15-245 Bialystok, Poland; ewaolch@uwb.edu.pl; 3 BioMedChem Doctoral School of the University of Lodz and Lodz Institutes of the Polish Academy of Sciences, Matejki 21/23, 90-237 Lodz, Poland; 4 Institute of Bioorganic Chemistry, Academy of Sciences of the Republic of Uzbekistan, Tashkent 100125, Uzbekistan; chemist.makhmudov@gmail.com (R.M.); rrakhimov.83@mail.ru (R.R.); nodiraabdulladjanova73@gmail.com (N.A.); 5 Faculty of Medicine, Collegium Medicum, Mazovian University in Plock, Pl. Dabrowskiego 2, 09-402 Plock, Poland; j.blasiak@mazowiecka.edu.pl; 6 Faculty of Health Sciences, Collegium Medicum, Mazovian University in Plock, Pl. Dabrowskiego 2, 09-402 Plock, Poland

**Keywords:** *Rhus glabra* L., *Pistacia vera* L., polyphenols, tannins, antiradical, antimicrobial activities

## Abstract

Various plant by-products have attracted increasing attention as sources of biologically active compounds, particularly polyphenols. In this study, tannin-rich polyphenolic fractions were isolated from the leaves of *Rhus glabra* L. and *Pistacia vera* L. (*Anacardiaceae*) using a simple and efficient extraction procedure. The chemical composition of the extracts was characterized by LC-MS analysis, and their antimicrobial, antiradical, and cytotoxic activities were evaluated. Both extracts exhibited strong antiradical activity against DPPH radicals, comparable to or exceeding that of vitamin C and Trolox. Furthermore, both extracts showed cytotoxic effects against HeLa cells while exerting virtually no effect on MRC-5 fibroblasts within the tested concentration range (1–50 µg/mL). Antibacterial activity was assessed against Gram-positive bacteria (*Staphylococcus aureus* ATCC 700699 (MRSA), *S. aureus* ATCC 8325-4 (MSSA), *Staphylococcus epidermidis* ATCC 51625 (MRSE), *S. epidermidis* ATCC 14990 (MSSE), *Streptococcus mutans* ATCC 25175, and *Bacillus cereus* ATCC 13061) and Gram-negative bacteria (*Escherichia coli* ATCC 35218 and *Pseudomonas aeruginosa* ATCC BAA-1744). The minimum inhibitory concentration (MIC) values ranged from 2 to 125 µg/mL for Gram-positive bacteria and from 125 to 500 µg/mL for Gram-negative bacteria. In all biological assays, the *R. glabra* extract showed greater activity than the *P. vera* extract, likely because of its higher flavonoid content despite similar tannin levels. Overall, the results indicate that these tannin-rich leaf extracts, particularly that of *R. glabra*, represent promising sources of bioactive compounds with potential practical applications. The possible contribution of synergistic interactions among extract constituents to the observed biological effects is also discussed.

## 1. Introduction

Plants are rich sources of phytochemicals with diverse biological properties. Among them, phenolic compounds, a large group of plant secondary metabolites, occupy a particularly important position due to their extensive use in traditional and folk medicine. Numerous studies have demonstrated the beneficial effects of polyphenols on human health, including their potential roles in preventing cancer, neurodegenerative disorders, and cardiovascular diseases, as well as in promoting longevity [1,2]. Phenolic compounds exhibit remarkable structural diversity, ranging from simple phenolic acids to highly complex tannins. These metabolites are distributed throughout virtually all plant organs and primarily serve protective functions against a broad spectrum of biotic stressors, including fungi, viruses, and bacteria, as well as abiotic factors such as ultraviolet (UV) radiation [3]. Depending on their chemical structure, phenolic compounds exhibit antioxidant and free-radical-scavenging properties and are capable of chelating metal ions, thereby contributing to the prevention of oxidative stress-related diseases [4].

Among plant polyphenols, tannins deserve particular attention. In addition to their antioxidant properties, tannins possess a unique ability to interact with proteins, including regulatory proteins, thereby influencing numerous physiological processes. Based on their chemical structure, tannins are currently classified into four major groups: condensed tannins, hydrolysable tannins (gallotannins and ellagitannins), phlorotannins, and complex tannins. In plants, these compounds are predominantly found in bark, roots, stems, leaves, and fruit peels [5,6].

In recent years, agri-food by-products have attracted considerable interest as sustainable and readily available sources of tannins and other bioactive compounds [7]. For example, tannin-rich fractions isolated from pistachio green hulls exhibit antioxidant and anti-lipase activities and have been proposed as potential alternatives to anti-obesity agents [8]. Tannins from pomegranate (*Punica granatum* L.) peels show antibacterial and anticancer properties [9], whereas tannins from *Pimenta dioica* leaves exhibit both antioxidant and anticancer activities [10]. Wild plants also represent valuable sources of tannins. For instance, tannins isolated from *Anabasis setifera* leaves have demonstrated antimicrobial, antioxidant, and anticancer potential [11]. Similarly, methanolic extracts from the leaves of *Momordica charantia*, *Senna podocarpa*, *Senna alata*, *Ocimum gratissimum*, and *Sida acuta* are rich in polyphenols and exhibit strong antimicrobial activity against a broad range of bacterial and fungal pathogens, including *Pseudomonas aeruginosa*, *Bacillus subtilis*, *Staphylococcus aureus*, *Klebsiella oxytoca*, *Proteus vulgaris*, *Enterobacter aerogenes*, *Aspergillus niger*, *Aspergillus flavus*, and *Candida albicans* [12]. Given their broad spectrum of biological activities, identifying new plant-derived tannin sources, optimizing extraction procedures, and evaluating their biological properties remain important objectives in the search for novel pharmaceuticals, food preservatives, and cosmetic ingredients.

The genus *Rhus*, comprising more than 250 species of flowering plants, has been used for centuries in culinary applications and traditional medicine. Its fruits are used to prepare a lemonade-like beverage and are also an ingredient of the popular spice mixture za’atar [13]. Preparations from various plant parts have traditionally been used to treat dysentery, diarrhea, stomatitis, dermatitis, and gynecological disorders, as well as for their anti-inflammatory and gastrointestinal benefits [14,15]. More recently, researchers have studied *Rhus* species because their extracts and isolated phytochemicals have shown antibacterial, antiviral, antidiabetic, and anticancer activities [16,17,18,19].

The genus *Pistacia* comprises approximately fifteen species, of which *Pistacia vera* L. is the only species cultivated commercially for edible nut production. Flavonoids are among the major phenolic constituents of pistachio nuts and contribute to their anti-inflammatory, antimicrobial, and antiviral properties [20]. Extracts from several *Pistacia* species have demonstrated significant cytotoxic activity against a variety of cancer cell lines, including those derived from lung, cervical, prostate, gastric, colon, liver, renal, skin, and breast cancers [21]. Polyphenol-rich leaf extracts of *Pistacia* species, including those with substantial tannin content, have also been reported to have antioxidant and antidiabetic activities [22,23].

In the present study, we describe a simple, efficient method for isolating tannin-rich polyphenolic fractions from the leaves of two members of the family *Anacardiaceae*, *Rhus glabra* L. and *Pistacia vera* L. We further characterized their chemical composition using LC-MS analysis and evaluated their antimicrobial, antioxidant, and cytotoxic activities in order to assess their potential as sources of biologically active compounds.

## 2. Results and Discussion

### 2.1. Extraction, Quantitative and Qualitative Analysis of Polyphenol Content in the Ethyl Acetate Fraction of R. glabra *L.* and P. vera *L.* Leaves

Crude extracts from *P. vera* L. and *R. glabra* L. leaves were obtained using 70% aqueous acetone, which has been reported to extract polyphenols from pistachio by-products more efficiently than 50% aqueous methanol or ethanol [24,25].

To obtain a polyphenol fraction enriched in tannins, we performed an additional extraction of the crude hydroacetone extracts with ethyl acetate. This fraction was then used for detailed phytochemical analysis and for determining biological activity. A diagram illustrating the isolation of a polyphenol fraction enriched with tannins is shown in Figure 7.

**Figure 1:**
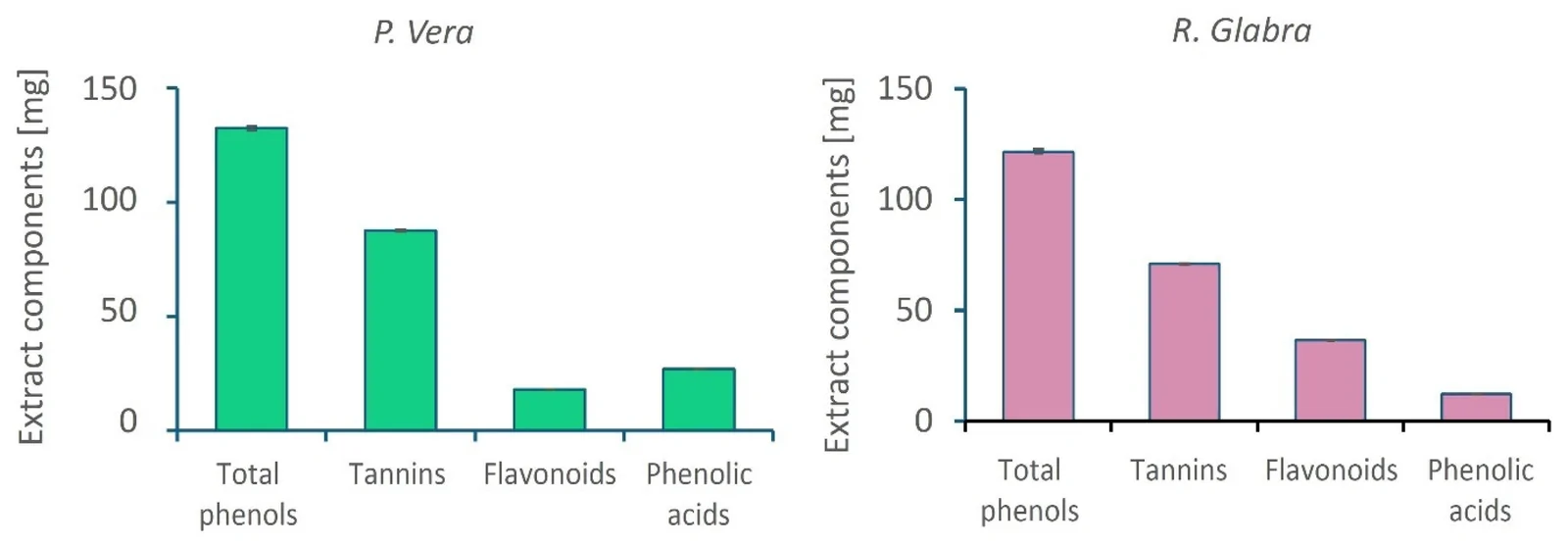
Quantitative polyphenol content (mg) in ethyl acetate extracts of *P. vera* L. and *R. glabra* L., expressed per 1 g of dry plant material.

The total polyphenol content of the ethyl acetate fraction was 121.40 ± 1.21 mg per 1 g of air-dry weight of raw material from *R. glabra* L. and 132.40 ± 1.14 mg per 1 g of air-dry weight of raw material from *P. vera* L. leaves (Figure 1). Following the preliminary separation of the total polyphenols from the ethyl acetate fraction of the two plants using hide powder, the total phenolic acids, flavonoids, and tannins were obtained.

As shown in the results, the total polyphenol content in both extracts is similar, at 12.14% and 13.24%, respectively, comparable to previously reported data (13.86%) for pistachio by-products [24]. Both extracts contain high levels of tannins, accounting for 59.0% (*R. glabra* L.) and 66.3% (*P. vera* L.) of the total polyphenol content, with the latter taken as 100%. However, the flavonoid content in the *R. glabra* L. extract was twice that in the *P. vera* L. extract, whereas the phenolic acid content was approximately 2.2 times lower (Figure 1). Hosseinzadeh’s study, which reported the polyphenol composition of different parts of the *P. vera* L. plant, also observed a high tannin content in the leaves and gum, in contrast to the fruits, where the flavonoid fraction was predominant [26]. Similarly, for other sumac species (*R. corriaria*, *R. typhina*) and *Pistacia* (*P. lentiscus*, *P. atlantica* Desf.), a high tannin content was observed in the leaves compared with other groups of polyphenols [27,28,29,30].

**Figure 2:**
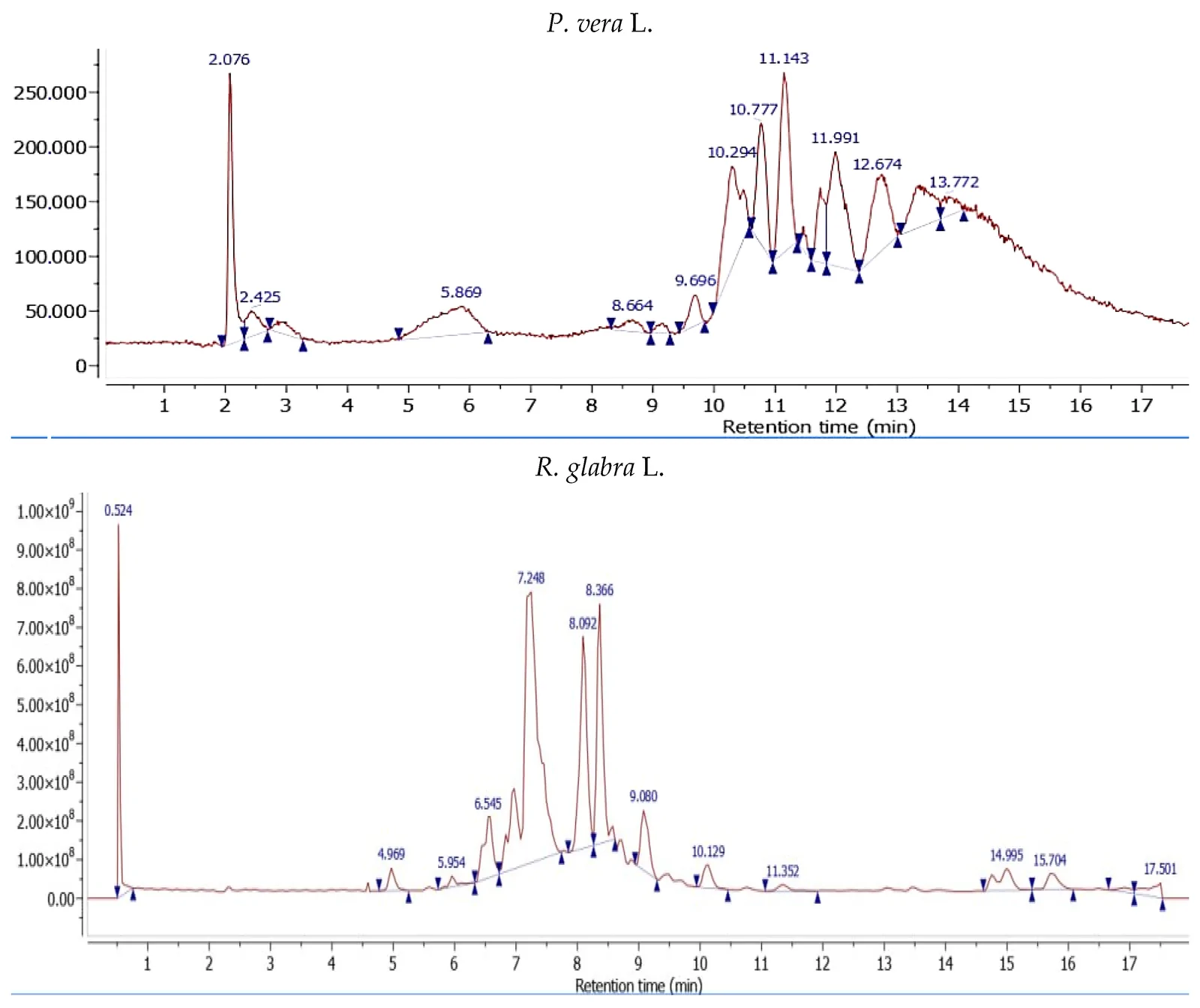
LC–MS base peak chromatogram of ethyl acetate fraction of *P. vera* L. (**top panel**) and *R. glabra* L. (**bottom panel**) in a negative ionization mode.

The detailed chemical composition of the extracts was subsequently investigated using LC–MS analysis. The spectra from this analysis are shown in Figure 2. Based on retention time, molecular weight, and fragmentation patterns, 21 compounds were identified in the ethyl acetate extract of *P. vera* L. (Table 1), and 30 compounds were identified in *R. glabra* L. (Table 2).

Hydrolysable tannins account for over 62% of the phenolic compounds in the ethyl acetate extract of *P. vera* L. leaves. Among them, gallic acid ethers of glucose include mono-, penta-, hexa-, hepta-, octa-, nona-, deca-, and nonadeca-galloyl glucose. Light hydrolysable tannins include galloyl shikimic acid, myricetin galloyl glucose, and quercetin galloyl glucose. Quinic acid, gallic acid, digallic acid, and trigallic acid make up 20%. Quercetin-glucoside, kaempferol-glucoside, myricetin-glucoside, (+)-catechin, and gallocatechin are representative flavonoids, totaling 13.5%.

Spectral analysis of the ethyl acetate fraction of *R. glabra* L. identified 30 polyphenols, of which 59.96% are hydrolyzable tannins—glucose derivatives substituted to varying degrees with gallic acid: di-, tri-, tetra-, penta-, hexa-, hepta-, octa-, and nonagalloyl glucose, and trigalloyl-levoglucosan. Gallic acid makes up 2.78%. Flavonoids and phenolic acids account for 35.3%: flavonoids include quercetin, luteolin, apigenin, kaempferol, isorhamnetin, and resveratrol (29.18%), and phenolic acids include protocatechuic acid, derivative valoneic, and caffeic acids (6.12%). Spectral analysis also identified substances such as 1,3-O-caffeoyl-dihydrocaffeoylglycerol, 1,5-di-O-galloyl-3,4-(S)-hexahydroxydiphenoyl protoquercitol, and rhaponticin, the content of which amounted to 2%.

Thus, the data obtained by LC–MS analysis are broadly consistent with the results obtained from separating the ethyl acetate extracts on a hide powder column (Figure 1). Specifically, despite having a similarly high tannin content (approximately 60%), the *R. glabra* extract contains a higher proportion of flavonoids and a lower proportion of phenolic acids than the *P. vera* L. extract.

### 2.2. Antioxidant and Cytotoxic Activities of the Ethyl Acetate Extracts Against Cancer and Non-Cancer Cells

#### 2.2.1. DPPH Scavenging Activity Assay

To evaluate the biological activity of ethyl acetate plant extracts, we focused on their antioxidant properties, as the compounds they contain, especially polyphenols, can neutralize free radicals and reactive oxygen species (ROS) implicated in the etiology of numerous diseases.

**Figure 3:**
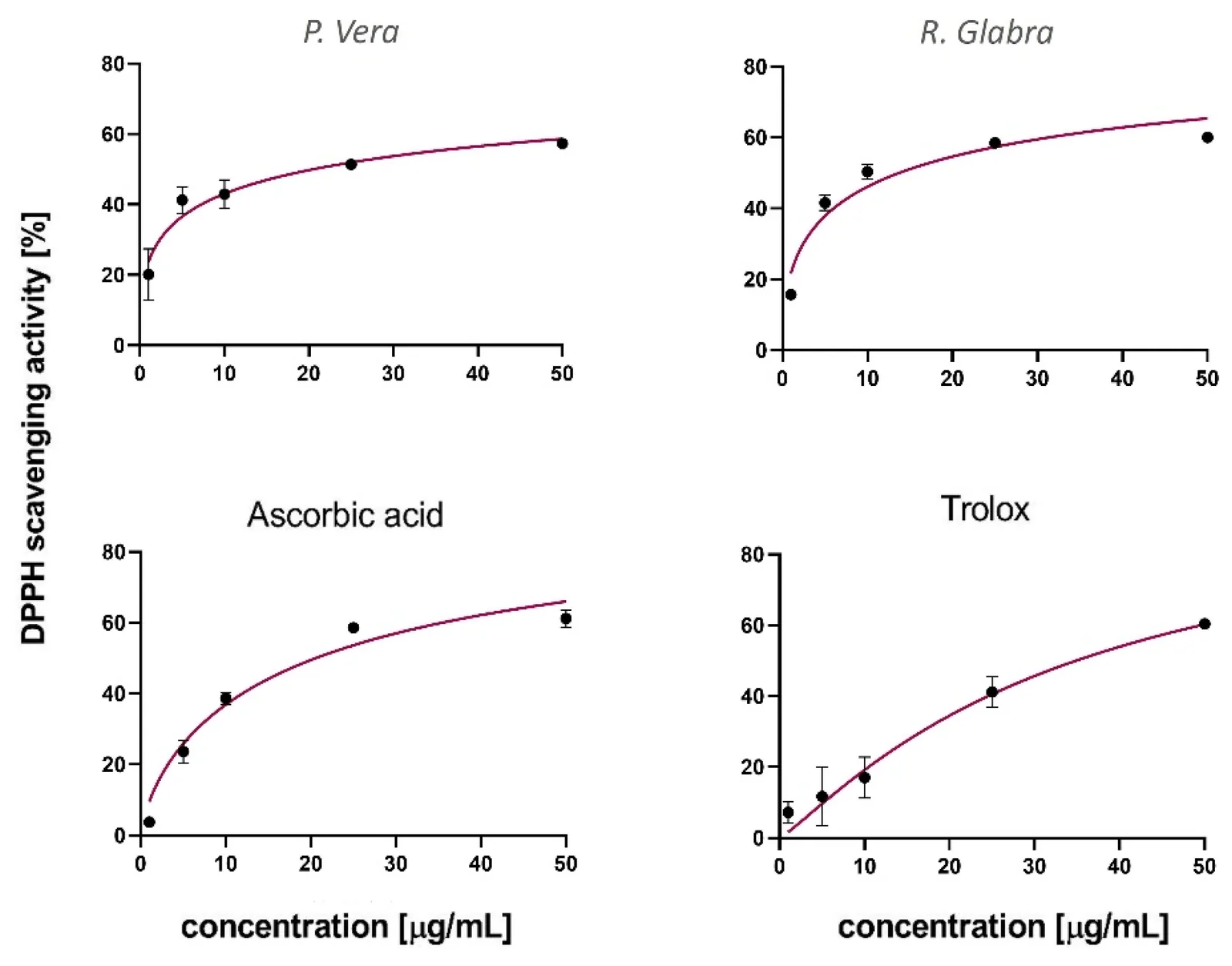
DPPH scavenging activity performed for tested ethyl acetate extracts in comparison to ascorbic acid and Trolox. DPPH was dissolved in ethanol and used in a final concentration of 0.25 mM. Graph represents mean values ± SD (*n* = 7). All tested extracts and reference antioxidants showed statistically significant, concentration-dependent DPPH scavenging activity compared to the control (*p* < 0.05).

In this study, the antioxidant activity of the tested plant extracts was evaluated using the DPPH radical-scavenging assay and compared with two reference antioxidants (ascorbic acid and Trolox) (Figure 3). Free radical-scavenging activity was expressed as the concentration of extract required to reduce 50% of the initial DPPH concentration (IC _50_). Results are presented in Table 3. Both extracts, *P. vera* L. and *R. glabra* L., exhibited a clear, concentration-dependent increase in DPPH-scavenging activity over the tested concentration range (1–50 µg/mL). At the lowest concentration, *P. vera* L. ethyl acetate extract demonstrated slightly higher radical-scavenging activity than *R. glabra* L., while both reference antioxidants showed only minimal effects. As concentration increased, antioxidant activity gradually rose across all tested samples. Notably, the IC _50_ values for both extracts were significantly lower than those of Trolox and ascorbic acid, indicating stronger antioxidant activity, consistent with previously reported data. Antov et al. reported that the aqueous methanolic extract of *Rhus typhina* leaves exhibited antioxidant activity in the DPPH assay, with an IC _50_ of 10 µg/mL [18]. Similarly, the ethyl acetate extract of *Rhus typhina* leaves exhibited strong DPPH radical-scavenging activity, with an IC _50_ of 2.12 µg/mL [27]. Furthermore, the hydroacetonic extract of *Pistacia lentiscus* leaves showed potent free-radical-scavenging activity in the DPPH assay, with an IC _50_ of 0.0027 mg/mL [22]. A comparative analysis of the IC _50_ values for the extracts showed that the IC _50_ value of *R. glabra* L. was 1.5 times lower than that of *P. vera*, indicating that the *R. glabra* L. extract exhibits stronger antioxidant activity. The observed difference in activity is likely related to variations in their chemical composition. As shown in Table 1, the tannin content in both extracts was comparable and accounted for more than 50% of the total polyphenol content, indicating a dominant role in the antioxidant activity of both extracts against DPPH. This assumption is supported by the findings of Sudirman et al., who demonstrated that the antiradical activity of *Pistacia stratiotes* leaf extract against DPPH increased during purification, which was accompanied by a parallel rise in tannin content [23].

Thus, the difference in the antioxidant activity of the studied extracts appears to be linked to differences in the content of flavonoids and phenolic acids. The *R. glabra* L. ethyl acetate extract contains twice as many flavonoids and half as many phenolic acids as *P. vera* L. The higher DPPH radical-scavenging activity of the *R. glabra* L. ethyl acetate extract can be attributed to its flavonoid content, as these compounds generally exhibit greater reactivity and electron-donating capacity than phenolic acids. It is known that the synergistic effect of the components in the extracts is not simply the sum of their radical-scavenging activities but also the result of their interactions [27]. It can be assumed that flavonoids may act as potent reducing agents for oxidized tannins because of their reaction with DPPH, thereby increasing the activity of the extracts.

#### 2.2.2. Cytotoxicity and Reactive Oxygen Species

Polyphenols, including tannins, can exert both antioxidant and pro-oxidant effects within cells. These dual properties depend on factors such as chemical structure, concentration, the surrounding microenvironment, pH, the presence of transition metals, their capacity to interact with macromolecules, and the specific cell type. Alterations in cellular redox potential induced by extracts rich in these compounds may lead to cytotoxic effects or, conversely, to enhanced cell proliferation [10,31,32]. Accordingly, we further evaluated the impact of these extracts on the viability of both non-cancerous (MRC-5) and cancer cells (HeLa) and conducted a parallel assessment of their intracellular ROS levels.

**Figure 4:**
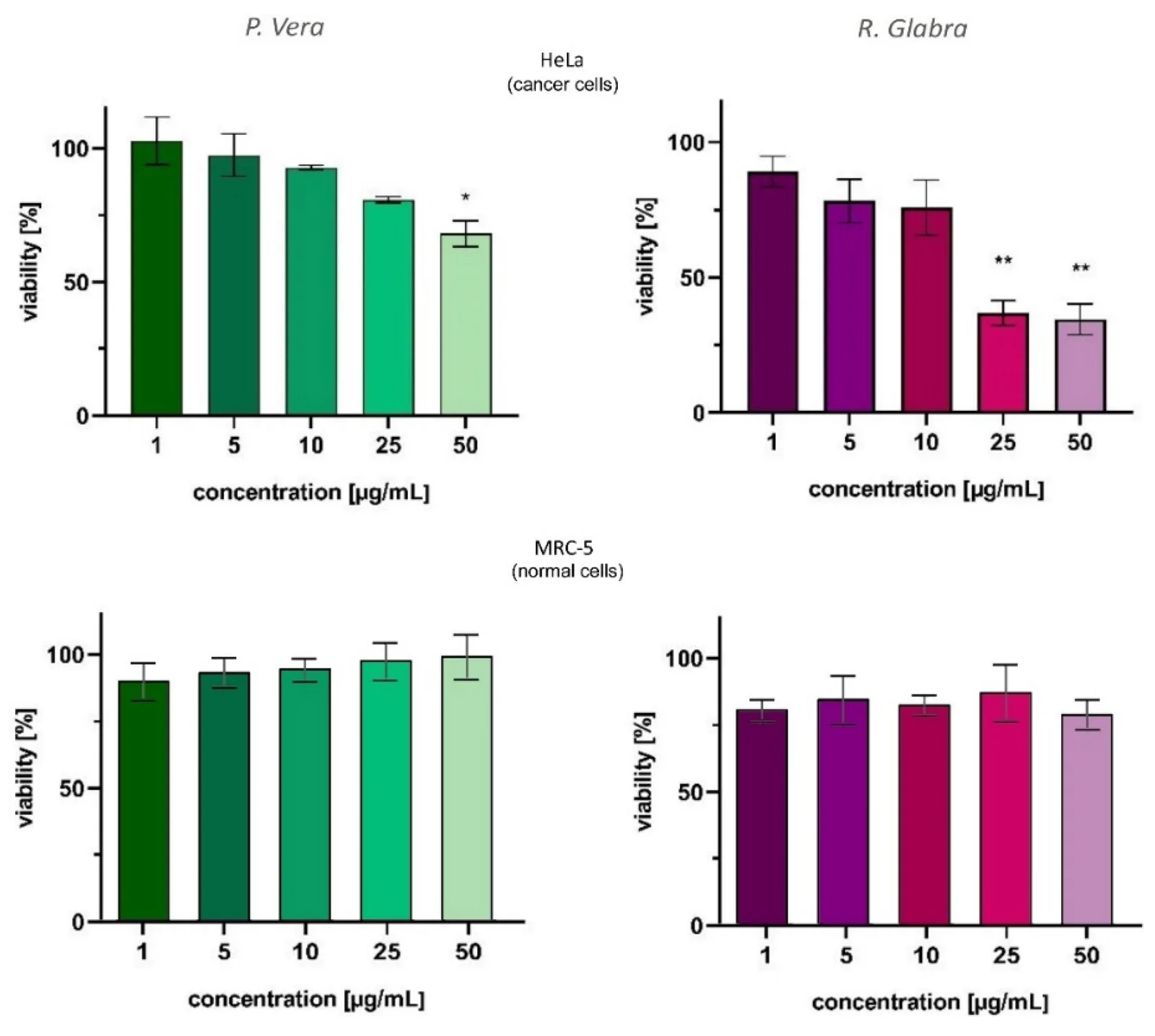
Cytotoxic effect of *P. vera* L. and *R. glabra* L. ethyl acetate extracts on HeLa (**top panel**) and MRC-5 (**bottom panel**) cell lines after 24 h incubation measured by MTT assay. Graph shows mean ± SD, *n* = 3. * *p* < 0.05 ** *p* < 0.005.

The cytotoxic effects of the tested ethyl acetate plant extracts were evaluated using the MTT assay in HeLa and MRC-5 cells over a concentration range of 1–50 µg/mL. In HeLa cells, treatment with *P. vera* L. extract produced a gradual, concentration-dependent decrease in cell viability (Figure 4). At lower concentrations, cell viability remained close to control values, indicating limited cytotoxicity. A moderate reduction in viability was observed at 25 µg/mL, while a statistically significant decrease was detected at the highest concentration tested (50 µg/mL). In contrast, the *R. glabra* L. ethyl acetate extract exhibited a more pronounced cytotoxic effect on HeLa cells. Although cell viability at lower concentrations remained above 70%, a marked, statistically significant reduction was observed at 25 and 50 µg/mL. At these higher concentrations, cell viability decreased to approximately 40% and 30%, respectively, indicating greater cytotoxic potential than the *P. vera* L. extract. Overall, the MTT results demonstrate a clear concentration-dependent cytotoxic response, with *R. glabra* showing higher cytotoxicity toward HeLa cells than *P. vera*. The IC_50_ for *P. vera* L. extract was 58.83 ± µg/mL, whereas that for *R. glabra* L. was 25.10 ± µg/mL.

The study of the cytotoxicity of the ethyl acetate extracts toward MRC-5 cells showed that, in contrast to the results for HeLa cells, they did not induce a significant reduction in MRC-5 viability across the tested concentration range. For the *P. vera* extract, MRC-5 viability remained close to or slightly above 100% at all concentrations, indicating the absence of cytotoxic effects and suggesting good cellular tolerance. Treatment of MRC-5 cells with the *R. glabra* extract slightly decreased viability, which, across the entire concentration range studied, did not exceed 20%.

**Figure 5:**
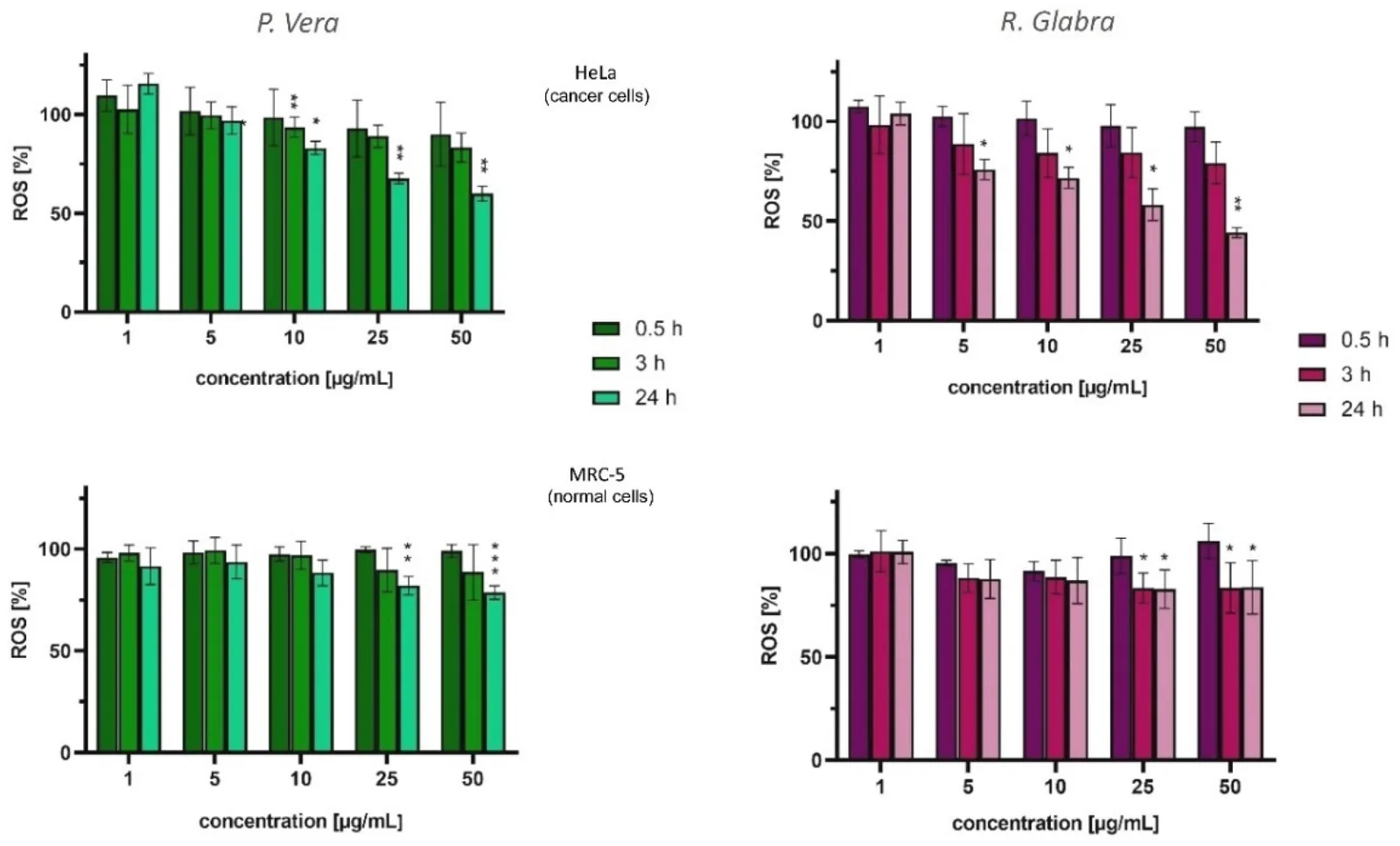
Reactive oxygen species formation in HeLa (**top panel**) and MRC-5 (**bottom panel**) cell lines after incubation with *P. vera* L. and *R. glabra* L. ethyl acetate extracts. Graph shows mean ± SD, *n* = 3, * *p* < 0.05 ** *p* < 0.005 *** *p* < 0.0005.

The effect of the tested ethyl acetate plant extracts on intracellular reactive oxygen species (ROS) levels in HeLa cells was evaluated after 0.5 h, 3 h, and 24 h of incubation at concentrations ranging from 1 to 50 µg/mL (Figure 5). Both *P. vera* L. and *R. glabra* L. ethyl acetate extracts induced concentration- and time-dependent changes in ROS production. For *P. vera*, no pronounced alterations in ROS levels were observed at the lowest concentrations (1–5 µg/mL) across incubation times, with values remaining close to those of the control. A gradual decrease in ROS generation became evident at higher concentrations (10–50 µg/mL), particularly after prolonged exposure. After 24 h of incubation, a statistically significant reduction in ROS levels was observed at the highest concentrations compared to the control, with an IC_50_ of 66.08 ± 11.57. In the case of *R. glabra* L., a more pronounced effect on intracellular ROS levels was detected. While short-term exposure (0.5 h) resulted in only minor changes, longer incubation times led to a marked and concentration-dependent decrease in ROS production. After 24 h, significant reductions in ROS levels were observed at 5 µg/mL, with the strongest effect detected at 50 µg/mL. The IC_50_ in this case was 32.43 ± 8.4. Overall, both ethyl acetate plant extracts modulated intracellular ROS levels in HeLa cells in a time- and concentration-dependent manner, with *R. glabra* L. exhibiting a stronger antioxidant effect than *P. vera*, particularly after prolonged incubation.

In MRC-5 cells treated with *P. vera* L. extract, ROS levels remained near control values at lower concentrations (1–10 µg/mL) across all tested time points. A noticeable reduction in ROS production was observed at higher concentrations (25–50 µg/mL). This effect was more pronounced after 24 h of incubation, with statistically significant decreases in ROS levels at 25 µg/mL and 50 µg/mL compared to the control. The reduction was moderate, not exceeding approximately 15–20% relative to untreated cells. In contrast, *R. glabra* L. extract exerted a stronger effect on intracellular ROS levels in MRC-5 cells. While short-term exposure (0.5 h) resulted in minimal or no significant changes, prolonged incubation (3 h and particularly 24 h) led to a clear concentration-dependent decrease in ROS production. After 24 h, statistically significant reductions were observed at 25 µg/mL and 50 µg/mL, with the most pronounced decrease recorded at the highest concentration.

In general, both ethyl acetate extracts showed antioxidant activity in MRC-5 cells, primarily at longer incubation times and higher concentrations. Consistent with observations in HeLa cells, *R. glabra* L. exhibited a more pronounced ROS-lowering effect than *P. vera*, although the overall modulation in MRC-5 cells was moderate and did not indicate excessive oxidative imbalance.

Comparison of both cell lines revealed that the *R. glabra* L. ethyl acetate extract exhibited a stronger antioxidant effect than the *P. vera* L. extract in both cell lines, while normal MRC-5 fibroblasts were less sensitive than cancerous HeLa cells. The data obtained are consistent with the anti-radical activity of the ethyl acetate extracts against DPPH, which also showed that the *P. glabra* L. extract is more potent (Table 4).

The obtained findings correlate with the effect of the ethyl acetate extracts on cell survival in both cancerous and normal cells; that is, a slight reduction in ROS levels in MRC-5 cells led to either no reduction in cell survival (as in the case of *P. vera* L.) or a slight reduction (as in the case of *R. glabra* L.). At the same time, in the case of Hela cells, a reduction in ROS levels led to a decrease in cell survival, with *R. glabra* L. extract proving more effective than *P. vera* L. in all experiments.

The difference in the effects of the two ethyl acetate extracts is likely due to variations in their chemical composition. As noted above, the *R. glabra* L. extract contains higher levels of tannins and flavonoids than *P. vera* L., which may enhance its biological activity and contribute to the observed differences in efficacy. A similar correlation between polyphenol content and anticancer activity has been demonstrated for aqueous leaf extracts from five *Cornus* L. species. All the extracts studied exhibited antiproliferative activity against MCF-7 cells, with the greatest effect observed in the extract with high tannin and flavonoid content [33].

The IC50 values we obtained for the cytotoxic activity of the extracts against HeLa cells are consistent with those reported in the literature. A water–methanol extract of *R. typhina* leaves, which exhibits high antioxidant activity against DPPH, inhibited the growth of the human breast cancer cell line MCF-7, with an IC50 of 56.8 µg/mL [18]. The ethyl acetate *Ansbasis setifera* leaf extract also demonstrated strong antitumor activity against Hep-G2 and MCF-7 cancer cells, with IC50 values of 36.4 and 44 µg/mL, respectively [11].

Polyphenols exhibit anti-cancer effects through complex, multi-targeted mechanisms, including modulation of key signaling pathways, alterations in lipid and energy metabolism, and induction of cell cycle arrest. These processes ultimately promote cancer cell death via apoptosis and autophagy [34,35]. Furthermore, one mechanism underlying the anti-cancer activity of polyphenols is modulation of the redox state of cancer cells. Although the literature mainly discusses the pro-oxidant activity of polyphenols as a mechanism underlying their anti-cancer activity [36,37], there is also evidence of a correlation between antioxidant activity and anti-cancer effects [38]. In particular, Grigalius et al. established a correlation between the anti-proliferative effect of trihydroxyflavones on A549 and U87 cell lines and their antioxidant activity [39]. The relationship between the antioxidant and anticancer activity of grape skin extracts against the human colon adenocarcinoma Caco2 cell line was also shown [40]. Cancer cells are characterized by an accelerated metabolism, which leads to elevated levels of reactive oxygen species (ROS). At the same time, ROS upregulate antioxidant defense systems to maintain a delicate oxidative balance that is essential for their survival and proliferation. In this context, a reduction in ROS levels by polyphenols may disrupt this finely tuned redox homeostasis, impair cancer cell metabolism, and ultimately induce cell death [38,41]. It appears that this mechanism underlies the anti-carcinogenic activity of the extracts studied.

### 2.3. Antimicrobial Activity of Ethyl Acetate Extracts from the Leaves of P. vera *L.* and R. glabra *L.*

In light of the growing resistance of bacteria to antibiotics, scientific research has focused on identifying alternative natural compounds. Polyphenols are known for their antioxidant activity and a wide range of biological effects, including antibacterial activity. Leaves of many plants are rich in polyphenols, especially tannins, which exhibit strong antibacterial properties and often surpass the effectiveness of conventional antibiotics. It has previously been demonstrated that a 95% ethanol extract derived from the leaves of *Voacanga globosa* Merr. exhibits broad-spectrum antimicrobial activity against several bacterial strains, including *Staphylococcus aureus*, *Bacillus cereus*, *Pseudomonas aeruginosa*, *Micrococcus luteus*, and *Salmonella typhimurium*, with activity greater than that of the standard antibiotic chloramphenicol [42]. The crude ethyl acetate extract of *Anabasis setifera* leaves, characterized by high concentrations of tannins, flavonoids, and alkaloids, demonstrated significant antimicrobial activity against a broad spectrum of bacterial strains. Results showed that the MICs of the extract against *E. coli*, *S. aureus*, *S. typhimurium*, and *B. subtilis* were 62.5, 125, 62.5, and 31.25 μg/mL, respectively [11]. The aqueous leaf extract of *Pistacia atlantica*, one of fifteen species of the genus *Pistacia*, showed strong antimicrobial activity against *Streptococcus mutans* (ATCC 35668) and *Streptococcus mitis* (ATCC 49456), with MIC values of 60 and 75 μg/mL, respectively [43].

In this study, we analyzed the antibacterial activity of acetyl acetate extracts from the leaves of *P. vera* L. and *R. glabra* L. against both Gram-positive bacteria (*S. aureus* ATCC 700699 (MRSA), *S. aureus* ATCC 8325-4 (MSSA), *S. epidermidis* ATCC 51625 (MRSE), *S. epidermidis* ATCC 14990 (MSSE), S. mutans ATCC 25175, B. cereus ATCC 13061) and Gram-negative bacteria (*E. coli* ATCC 35218, *P. aeruginosa* ATCC BAA-1744) by determining two parameters: MIC and MBC. As shown in Table 4, MIC values for the extracts against Gram-positive bacteria ranged from 2 to 125 µg/mL, depending on the bacterial strain. The most sensitive strain to both extracts was *S. epidermidis* ATCC 14990 (MSSE). For Gram-negative bacteria, MIC values were two times higher, ranging from 125 to 500 µg/mL. The same trend was observed for the MBC. For Gram-positive bacteria, the range was 31.25–500 µg/mL, whereas for Gram-negative bacteria it was significantly higher, at 1000–2000 µg/mL (Table 4).

The data indicate that both ethyl acetate extracts were more effective against Gram-positive than against Gram-negative bacteria, consistent with the literature. A 70% *v*/*v* ethanol crude leaf extract of *P. vera* L. has previously been shown to exhibit antimicrobial activity against *S. aureus* and *K. pneumoniae*, with MICs ranging from 0.60 to 0.10 mg/mL, whereas *P. aeruginosa* was the least sensitive bacterium to the extract [44]. The aqueous crude extract of *P. vera* leaves, which contains numerous flavonoids, including galloyl and glycosyl derivatives of quercetin and myricetin, exhibited high activity against several bacteria, including *S. aureus*, *E. faecalis*, *K. pneumoniae*, and *S. agalactiae*, with the exception of *E. coli* [45].

A comparison of the antimicrobial activity of the ethyl acetate extracts with that of the β-lactam antibiotic oxacillin showed that both extracts were more effective against Gram-negative bacteria than oxacillin. Among Gram-positive bacteria, the *R. glabra* extract exhibited significantly greater antimicrobial activity against *S. aureus* ATCC 700699 (MRSA), *S. epidermidis* ATCC 51625 (MRSE), *S. mutans* ATCC 25175, and *B. cereus* ATCC 13061. In contrast, the *P. vera* L. extract showed significantly greater activity than oxacillin only against *S. aureus* ATCC 700699 (MRSA) and *S. epidermidis* ATCC 51625 (MRSE).

A comparative analysis of the activity of the ethyl acetate leaf extracts under study showed that the *R. glabra* L. extract was twice as effective as the *P. vera* L. extract against both Gram-positive and Gram-negative bacteria. This difference appears to be linked to the extracts’ distinct chemical compositions, specifically the higher levels of tannins and flavonoids in the *R. glabra* L. extract compared with the *P. vera* L. extract. Our hypothesis is supported by data from other authors. Previous studies have demonstrated that crude methanolic extracts of the leaves of five plants from different families exhibited marked antimicrobial activity against clinical isolates, including both Gram-positive (*Staphylococcus aureus*, *Bacillus subtilis*) and Gram-negative (*Klebsiella oxytoca*, *Pseudomonas aeruginosa*, *Proteus vulgaris*, *Enterobacter aerogenes*) bacteria, with MIC values ranging from 31.25 to 250 mg/mL. The most active extracts were those containing tannins and flavonoids, which, according to the authors, demonstrate a synergistic interaction that enhances their biological activity [12]. The antibacterial activity of a 60% (*v*/*v*) ethanol crude leaf extract of *Rhus coriaria* and its subfractions was evaluated against *Klebsiella pneumoniae*, *Escherichia coli*, *Bacillus subtilis*, and *Staphylococcus aureus*. The extract demonstrated measurable antibacterial effects against all tested strains. The MIC ranged from 0.2 to 3.1 mg/mL, and the MBC from 0.3 to 6.2 mg/mL, depending on the bacterial species and the fraction tested. Among the subfractions, the one rich in flavonoids and tannins showed the strongest antibacterial activity, with an MIC of 0.4 mg/mL [30].

The mechanism of antimicrobial activity of the ethyl acetate extracts of *R. glabra* L. and *P. vera* L., as shown in this work, is unclear and may be multifaceted. Polyphenols can act on whole bacteria by damaging or modifying membrane structure, inhibiting energy metabolism, reducing the production or secretion of virulence factors, and preventing biofilm formation [16]. The tested extracts contain more than 50% tannins, which strongly interact with proteins. These compounds can bind key proteins involved in bacterial cellular metabolism, thereby impairing viability. Furthermore, the flavonoids and tannins present in the extract can induce oxidative stress in bacterial cells. The mechanism underlying ROS formation may be linked to the effects of extract components on bacterial cell signaling or to the auto-oxidation of polyphenols [9]. It is also possible that the extract components may induce changes in the structural organization of bacterial cell membranes. We previously demonstrated that an ethyl acetate extract of *R. typhina* leaves, containing a high concentration of tannins, exhibited greater antibacterial activity against *Bacillus cereus* than against *Pseudomonas aeruginosa*. This difference was related to membrane-targeting effects: specifically, decreased membrane fluidity in B. cereus and only mild membrane disruption in *P. aeruginosa* [46].

In summary, our data indicate that ethyl acetate extracts from the leaves of *R. glabra* L. and *P. vera* L. may serve as a source of potent bioactive compounds for developing antimicrobial agents.

## 3. Materials and Methods

### 3.1. Plant Material

**Figure 6:**
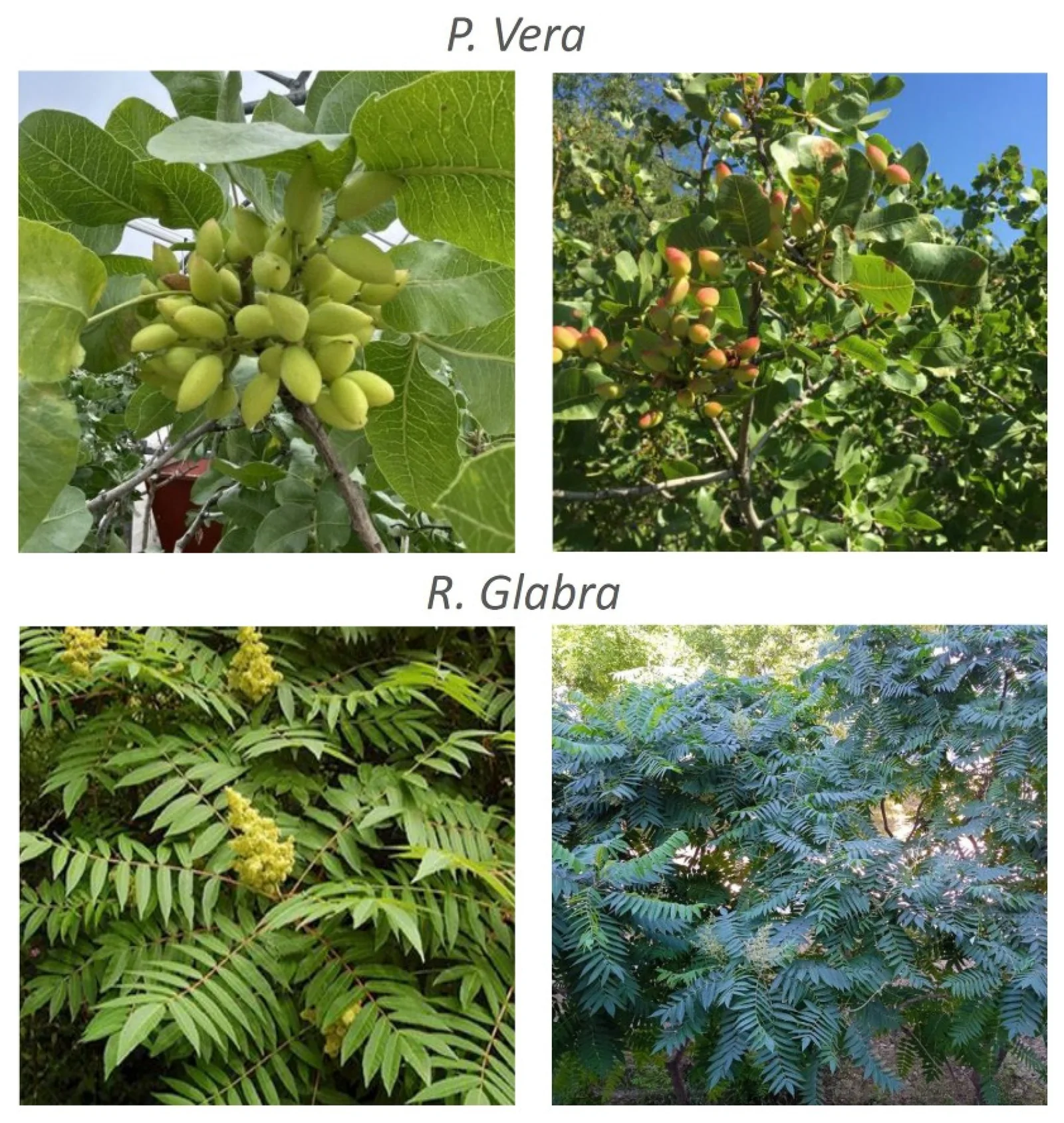
Representative plants of *Pistacia vera* L. and *Rhus glabra* L. and their parts. Location—Uch-terak village, Boʻstonliq District, Tashkent Region (Chimgan Mountains), Uzbekistan. *Pistacia vera* L. is deposited in the National Herbarium of Uzbekistan under accession number 100720218.1. *Rhus glabra* L. is deposited in the National Herbarium of Uzbekistan under accession number 06.

Specimens of *Pistacia vera* L. and *Rhus glabra* L. were collected in Uch-terak village, Boʻstonliq District, Tashkent Region (Chimgan Mountains), Uzbekistan (Figure 6). The collection site is located at 41.533866° N and 69.914925° E, at an elevation of 1248 m above sea level. *Pistacia vera* L. is deposited in the National Herbarium of Uzbekistan under accession number 100720218.1 and identified by K. Sh. Tojibaev. *Rhus glabra* L. is deposited in the National Herbarium of Uzbekistan under accession number 06 and identified by O.T. Turginov.

### 3.2. Preparation of the Ethyl Acetate Extract of P. vera *L.* and R. glabra *L.* Leaves

**Figure 7:**
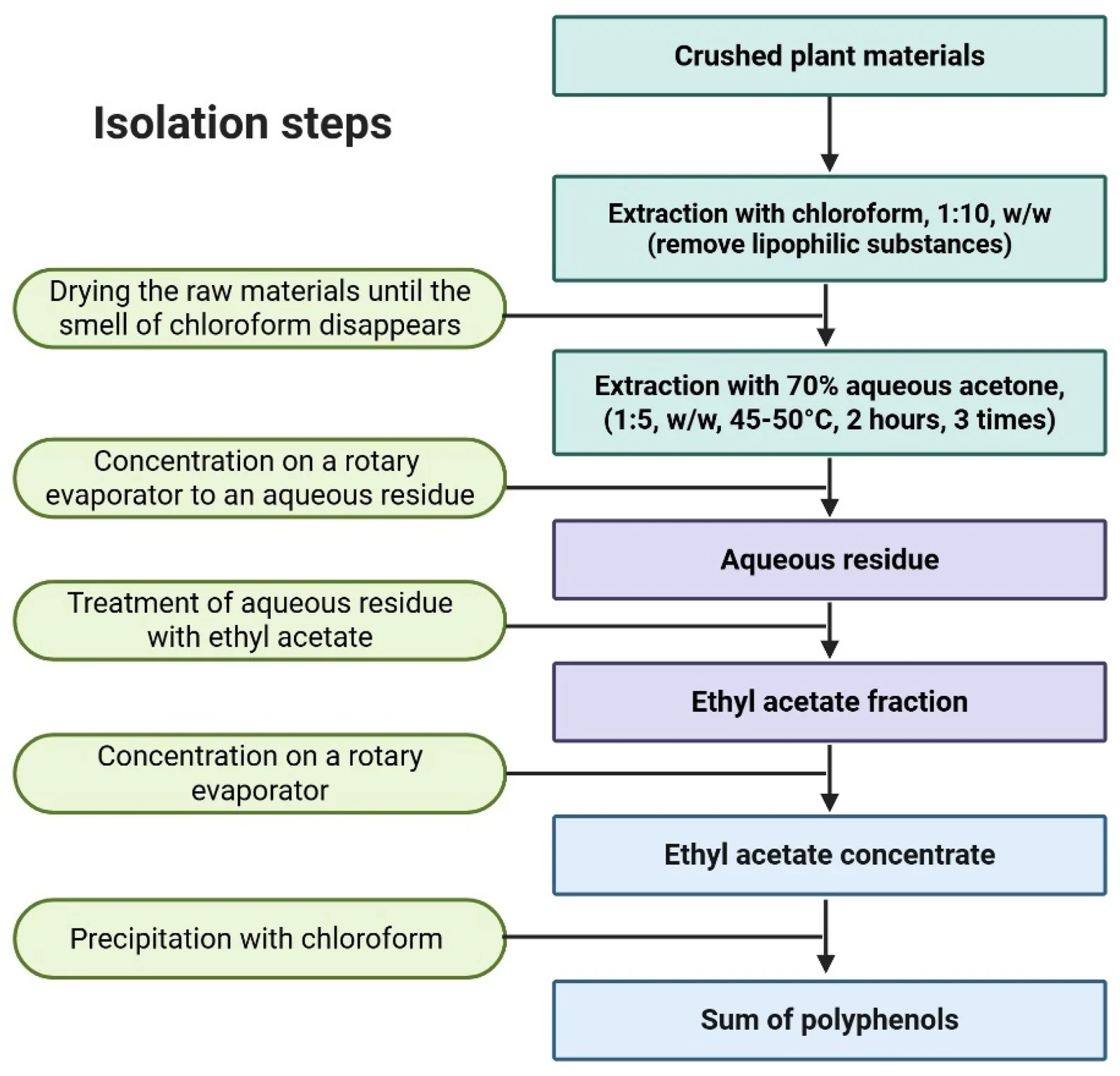
Schematic diagram of the isolation of the ethyl acetate fraction sum of polyphenols.

The ethyl acetate fractions were prepared as described [47], with modifications. Air-dried, crushed (2–4 mm) leaves of *R. glabra* L. and *P. vera* L. (100 g) were pre-extracted with chloroform to remove lipophilic substances. For this purpose, the raw materials were placed in a 3 L flask equipped with a reflux condenser and extracted with chloroform in a water bath at a 1 g:10 mL (raw materials:chloroform) ratio for 2 h, followed by three additional extractions. The raw materials were then dried at room temperature. Air-dried, defatted materials were extracted with 70% aqueous acetone at a 1 g:5 mL ratio. The extraction was repeated three times. Next, the resulting water–acetone extracts were evaporated under vacuum on a rotary evaporator until the aqueous portion remained. The aqueous residue was further treated with chloroform (1:4, *v*/*v*). The chloroform fraction was discarded, and the aqueous fraction was repeatedly extracted (3 times) with ethyl acetate (1:5, *v*/*v*). The resulting ethyl acetate fraction was dried over anhydrous Na_2_SO_4_, concentrated to a small volume, and polyphenols were precipitated by adding three volumes of dry chloroform. The polyphenol precipitate was filtered through a Büchner funnel with filter paper, washed with dry chloroform, and then dried in a vacuum oven at room temperature. The extract yield was determined by the gravimetric method. A schematic diagram of the isolation of the ethyl acetate extracts is shown in Figure 7.

### 3.3. Determination of Phenolic Acid, Flavonoids and Tannins Content in Ethyl Acetate Fractions

The contents of the various classes of phenolic compounds were determined after separating the ethyl acetate extracts into fractions by column chromatography on hide powder, according to [48,49]. The ethyl acetate fractions obtained from *R. glabra* L. (12 g) and *P. vera* L. (13 g) were dissolved in 200 mL of distilled water at 40 °C, cooled to room temperature, and then diluted to a final volume of 500 mL with distilled water.

Hide powder (100 g) was suspended in 1000 mL of distilled water and agitated on a shaker for 20 min. The powder was then separated by squeezing and mixed with 500 mL of the prepared polyphenol solution. The mixture was agitated on a shaker for an additional 45 min. The hide powder containing the adsorbed compounds was subsequently packed into a chromatographic column (4.5 × 145 cm). The column was washed sequentially with diethyl ether, water, and 60% aqueous acetone until the phenolic-compound test was negative. The elution process was monitored by thin-layer chromatography (TLC) using a toluene–acetone–formic acid solvent system (6:6:1). Qualitative tests using ammonia vapor, Na_2_CO_3_ solution, and 1% ethanolic FeCl_3_ solution indicated that the phenolic compounds present in the diethyl ether fraction were phenolic acids, those in the aqueous fraction were flavonoids, and those in the 60% aqueous acetone fraction were tannins [48].

$$X\;[\%]=\frac{\mathrm{mass}\;\mathrm{of}\;\mathrm{individual}\;\mathrm{fractions}\;\left(g\right)}{\mathrm{mass}\;\mathrm{of}\;\mathrm{the}\;\mathrm{ethyl}\;\mathrm{acetate}\;\mathrm{fraction}\;\left(g\right)}\times 100$$

### 3.4. LC-MS Study of Polyphenols

Mass spectrometric studies of isolated polyphenols from *P. vera* L were conducted on an Agilent Technologies 6520B series Q-TOF LC-MS instrument (Agilent Technologies, Santa Clara, CA, USA)controlled by MassHunter Software, Data Acquisition, Version B.05.00.Computer software. (Santa Clara, CA, USA, Agilent Technologies, 2011) under the following conditions: ionization source—ESI, drying gas flow—5 L/min, drying gas temperature—300 °C, voltage at: skimmer cone—20 V, fragmentor—125 V, mass range: MS 100–2000 *m*/*z*, collision energy—35, 50 eV. Ionization method: negative. Samples were introduced into the mass spectrometer using an Agilent Technologies 1200 series HPLC System. (Agilent Technologies, Santa Clara, CA, USA) chromatograph with a Zorbax SBC18 column (3 µm, 0.5 × 150 mm). Mobile phase: A—0.1% formic acid solution, B—acetonitrile containing 0.1% formic acid. Elution was performed on an Agilent Technologies 1260 Series Infinity Capillary Pump instrument (Agilent Technologies, Santa Clara, CA, USA). Cap Pump instrument at a flow rate of 15 µL/min. Gradient of solution B concentration (minutes): 0–5 min—20%, 5–20 min—25%, 20–25 min—30%, 25–30 min—60%, 30–35 min—20%. Solutions were degassed using an Agilent Technologies 1260 µ-degasser. Samples were applied to the column using an Agilent Technologies Micro WPS device, 1 µL each, from a polyphenol solution at 0.1 mg/mL. Detection wavelength: 269 nm.

The polyphenolic compounds in the *R. glabra* L. extract were analyzed using a UHPLC-Q-Orbitrap-HRMS system (Thermo Fisher Scientific, Bremen, Germany). HRMS/MS detection was performed in negative ion mode over a mass range of *m*/*z* 100–2000 at a resolution of 70,000 (FWHM), with the resolving power specified at *m*/*z* 200. Ion source parameters were set as follows: spray voltage, 3.5 kV; heater temperature, 350 °C; and capillary temperature, 350 °C. The sheath gas and auxiliary gas flow rates were 40 and 10 arbitrary units (arb), respectively. The normalized collision energy (NCE) was set to 40.

Chromatographic separation was performed on a Thermo Scientific Acclaim VANQUISH C18 column (2.2 μm, 2.1 × 150 mm) at 30 °C. The mobile phase consisted of (A) 0.1% formic acid in water and (B) acetonitrile with 0.1% formic acid. The gradient for mobile phase B was as follows: 0–5 min, 10%; 6–15 min, 60%; 16–18 min, 80%; and 20–30 min, 20%. The flow rate was 0.3 mL/min. The detection wavelength was set to 280 nm.

Identification was performed by interpreting MS/MS spectra in comparison with data reported in the literature. During the identification process, the following public databases were consulted: Chemical Entities of Biological Interest (ChEBI, www.ebi.ac.uk/chebi), Chemical Compounds Deep Data Source (https://www.molinstincts.com), ChemSpider (www.chemspider.com), and Phenol_Explorer (www.phenol-explorer.eu).

Because the fractions studied consist predominantly of phenolic compounds with similar chemical properties and comparable ionization efficiencies, the peak area in the integrated total ion current (TIC) chromatogram is assumed to be proportional to the molar percentage of each component [50]. The mass percentage of each component was estimated from its peak area in the LC–MS TIC chromatogram using the following formula:

$$\omega_{i}\left[\%\right]=\frac{M_{i}}{\sum_{j=1}^{n}\;\left(x_{i}\cdot M_{i}\right)}\times 100$$

where $\omega_{i}$ (%) is the mass percentage of component *i*; $x_{i}$ is the molar fraction of component *i*, calculated from its peak area in the LC–MS data (peak area/total peak area); $M_{i}$ is the molar mass of component *i*; and $\sum (\left.x_{j}M_{j}\right)$ is the sum of the products of the molar fractions and corresponding molar masses of all identified components.

### 3.5. DPPH Radical Scavenging Assay

The antioxidant activity of the tested ethyl acetate extracts was determined using the DPPH (2,2-diphenyl-1-picrylhydrazyl) radical-scavenging assay and compared with the reference antioxidants, ascorbic acid and Trolox. A DPPH solution was prepared in ethanol and used at a final concentration of 0.25 mM. The ethyl acetate extracts and reference compounds were tested over a concentration range of 1–50 µg/mL.

For the assay, 180 µL of the DPPH solution was mixed with 20 µL of the tested sample in a 96-well microplate. The reaction mixtures were incubated for 30 min at room temperature in the dark. Following incubation, the decrease in absorbance was measured at 517 nm using a microplate reader. DPPH solution was used as a control (100%).

The DPPH radical scavenging activity was calculated as the percentage of reduction according to the following equation:

$$\mathrm{DPPH}\;\mathrm{scavenging}\;\mathrm{activity}\;(\%)=\left(\frac{A_{0}-A_{s}}{A_{0}}\right)\times 100$$

where $A_{0}$ is the absorbance of the control and $A_{s}$ is the absorbance of the reaction mixture containing the tested extract or reference antioxidant. All measurements were performed in triplicate, and the results are presented as mean ± standard deviation [51].

### 3.6. Reactive Oxygen Species Determination

The human lung fibroblast cell line (MRC-5) and the human cervical carcinoma cell line (HeLa) were used for in vitro studies. Both cell lines were obtained from the American Type Culture Collection (ATCC, Manassas, VA, USA). Cells were maintained in DMEM (Gibco, Thermo Fisher Scientific, Waltham, MA, USA) supplemented with 10% heat-inactivated FBS and 1% penicillin/streptomycin. Cells were cultured in plastic tissue culture flasks (Falcon, GE Healthcare Life Sciences, Chicago, IL, USA) at 37 °C in a humidified atmosphere of 5% CO_2_ and 95% air.

Intracellular generation of reactive oxygen species (ROS) in HeLa and MRC-5 cells after exposure to ethyl acetate plant extracts was measured using a fluorometric assay based on 2′,7′-dichlorodihydrofluorescein diacetate (H_2_DCF-DA; Thermo Fisher Scientific, Waltham, MA, USA). This non-fluorescent probe readily penetrates cell membranes and is deacetylated by intracellular esterases to form DCFH. Oxidation of DCFH by ROS yields the fluorescent compound dichlorofluorescein (DCF), enabling quantitative assessment of ROS levels.

HeLa cells were plated in black 96-well microplates at 10^4^ cells per well and maintained under standard culture conditions. After 24 h of incubation, cells were treated with ethyl acetate extracts from *R. glabra* and *P. vera*. ROS production was assessed at 0.5 h, 3 h, and 24 h post-exposure. After treatment, cells were rinsed with phosphate-buffered saline (PBS) and incubated with H_2_DCF-DA at 2 µmol/L for 15 min at 37 °C. Fluorescence was measured using a microplate reader (BioTek PowerWave HT, BioTek Instruments, Inc., Winooski, VT, USA) with excitation and emission wavelengths set to 485 nm and 530 nm, respectively [51].

### 3.7. Cell Viability Assay (MTT)

The cytotoxic effects of ethyl acetate plant extracts on HeLa cells were evaluated using the MTT colorimetric assay. Cells were plated in 96-well culture plates at 1 × 10^4^ cells per well and allowed to adhere under standard incubation conditions. After 24 h, the culture medium was replaced with fresh medium containing ethyl acetate plant extracts at concentrations ranging from 1 to 50 µg/mL, followed by an additional 24 h incubation.

Subsequently, an MTT solution prepared in PBS (final concentration 0.5 mg/mL) was added to each well, and the plates were incubated for 3 h to allow formazan crystal formation. The reaction medium was then removed, and 100 µL of DMSO was added to solubilize the crystals. Absorbance was measured at 580 nm with background correction at 720 nm using a multiwell plate reader (BioTek PowerWave HT, BioTek Instruments, Inc., Winooski, VT, USA).

Cell viability was expressed as a percentage relative to untreated control cells and calculated according to the following equation:

$$\mathrm{Cell}\;\mathrm{viability}\;[\%]=\left(\frac{A_{s}}{A_{c}}\right)\times 100$$

where $A_{s}$ represents the absorbance of extract-treated cells and $A_{c}$ corresponds to the absorbance of control cells [52].

### 3.8. Antimicrobial Activity—Determination of Minimum Inhibitory Concentration (MIC) and Minimum Bactericidal Concentration (MBC)

The antibacterial activity of ethyl acetate extracts was assessed by monitoring the growth of eight bacterial strains (*Staphylococcus aureus* ATCC 700699, *Staphylococcus aureus* 8325–4, *Staphylococcus epidermidis* ATCC 51625, *Staphylococcus epidermidis* ATCC 14990, *Streptococcus mutans* ATCC 25175, *Bacillus cereus* ATCC 13061, *Escherichia coli* ATCC 35218, and *Pseudomonas aeruginosa* ATCC BAA-1744) using the broth microdilution method, conducted according to the National Committee for Clinical Laboratory Standards [53].

The reference bacterial strains were obtained from the ATCC collection (Microbiologics, MN, USA). All microorganisms were stored at −80 °C in a storage medium consisting of LB broth and glycerol (1:1). They were then inoculated onto nutrient agar and incubated overnight at 37 °C. The β-lactam antibiotic oxacillin served as the reference standard.

First, ethyl acetate extracts were dissolved in water (8000 µg/mL), and the solution was added to Mueller-Hinton broth. Next, the samples were serially twofold diluted in bullion to obtain concentrations ranging from 4000 to 0.976 µg/mL in a 96-well microtiter plate with a final volume of 100 µL. Then, 100 µL of bacterial solution was added to each well. The final bacterial cell concentration was 1 × 10^6^ colony-forming units per milliliter (CFU/mL), while the final concentrations of the test extracts in the 96-well plates ranged from 2000 to 0.488 µg/mL. The plates were incubated at 37 °C for 24 h. The MIC was defined as the lowest concentration of an antibacterial agent required to inhibit bacterial growth, as indicated by the absence of turbidity. Five microliters of the overnight culture from each well in the plate containing tested extracts at concentrations equal to or higher than the MIC value were transferred onto MH agar and incubated overnight at 37 °C. The MBC value was the lowest concentration at which no bacterial growth was observed on nutrient agar [54].

### 3.9. Data Analysis

Two-way ANOVA was performed with at least three independent replicates. We used Dunnett’s multiple-comparison test to evaluate effects within the data rows. IC_50_ values were estimated by fitting the dose–response data to a standard logistic equation using GraphPad Prism, version Prism 10.3.1. for Windows (graphPad softweare, Boston, MA, USA).

## 4. Conclusions

In conclusion, the present study demonstrated that the leaves of *Rhus glabra* L. and *Pistacia vera* L. are valuable sources of tannin-rich polyphenolic fractions with significant biological activity. Both ethyl acetate extracts contained high levels of hydrolyzable tannins, along with flavonoids and phenolic acids, although their chemical composition and biological activity differed notably. The *R. glabra* L. extract contained higher levels of flavonoids and exhibited stronger antioxidant, antimicrobial, and anticancer activities than the *P. vera* L. extract, highlighting the crucial role of other polyphenolic constituents in enhancing the bioactivity of tannin-rich extracts.

The ethyl acetate extracts exhibited strong DPPH radical-scavenging capacity and markedly reduced intracellular ROS levels, particularly in HeLa cancer cells. Notably, *R. glabra* L. displayed selective cytotoxicity against cancer cells, with only limited effects on normal fibroblasts. These extracts also showed substantial antibacterial activity against a broad range of bacterial species, in some cases exceeding that of the reference β-lactam antibiotic oxacillin, particularly against Gram-negative bacteria. The strain most sensitive to both extracts was *Staphylococcus epidermidis* ATCC 14990, suggesting the potential use of these extracts in treating skin infections. Overall, the results indicate that the tannin-rich leaf extracts studied, mainly *R. glabra* L., may be promising candidates for developing natural antioxidant, antimicrobial, and anticancer substances. Further studies focused on identifying active compounds and elucidating their molecular mechanisms of action are warranted.

## Figures and Tables

**Table 1:** Phenolic compounds detected and identified in ethyl acetate fraction of *P. vera* L. leaves by LC-QTOF-MS.

R_t_, min	Identification	Content, %	[M-H]^−^, *m*/*z*	Molecular Formula (Error, ppm)	MS/MS, *m*/*z* (Intensity, %)
1.95	Quinic acid	1.69	191.0569	C_7_H_12_O_6(4.2)_	171.0442 (12), 137.0302 (10), 127.0034 (100)
1.95	Digalloyl shikimic acid	1.85	477.0658	C_21_H_18_O_13(−3.4)_	325.0148 (100), 169.0102 (47), 125.0425 (11)
2.03	Galloyl glucose	2.98	331.0680	C_13_H_16_O_10(2.8)_	169.0144 (36), 137.0506 (100), 125.0191 (7)
2.08	Gallic acid	9.49	169.0138	C_7_H_6_O_5(−2.5)_	125.0286 (100)
2.08	Galloyl shikimic acid	2.42	325.0590	C_14_H_14_O_9(7.7)_	281.0191 (7), 169.0152 (100), 125.0339 (15)
2.97	(+)-Gallocatechin	2.21	305.0640	C_15_H_14_O_7(−8.7)_	197.0144 (100), 179.0278 (22), 169.0153 (21), 137.0291 (32)
5.85	(+)-Catechin	2.15	289.0730	C_15_H_14_O_6(4.3)_	271.0581 (100), 245.0102 (14), 221.0252 (10), 163.0425 (4), 151.0371 (18), 137.0404 (18)
8.53	Digallic acid	3.28	321.0245	C_14_H_10_O_9(−2.1)_	169.0149 (100), 125.0664 (16)
9.11	Myricetin galloyl glucoside	1.60	631.0920	C_28_H_24_O_17(−3.2)_	479.0784 (53), 317.0699 (25), 137.0572 (100), 112.0184 (31)
9.68	Myricetin glucoside	1.67	479.0843	C_21_H_20_O_13(2.5)_	316.0183 (100), 179.0618 (6), 271.0187 (12)
10.14	Trigallic acid	5.54	473.0355	C_21_H_14_O_13(−1.3)_	321.0453 (100), 169.0485 (25), 125.0140 (6)
10.29	Quercetin-galloyl-glucoside	4.12	615.1013	C_28_H_24_O_16(3.5)_	301.0705 (100), 463.0772 (34)
10.78	Quercetin-glucoside	5.64	463.0878	C_21_H_20_O_12(−0.8)_	301.0524 (100); 300.0218 (75); 271.0292 (5); 179.0054 (5)
11.16	Pentagalloyl glucose	9.08	939.1094	C_41_H_32_O_26(−1.5)_	787.0352 (10), 615.2734 (3), 483.2187 (17), 469.3483 (100), 331.2781 (4), 169.0197 (5)
11.74	Kaempferol-glucoside	1.86	447.0952	C_21_H_20_O_11(4.3)_	285.2454 (100), 198.0756 (10), 163.0743 (7), 151.0970 (5)
11.99	Hexagalloyl-glucoside	8.39	1091.1186	C_48_ H_36_O_30(−2.9)_	939.0768 (4), 787.1180 (4), 635.0153 (100), 331.0678 (2), 295.0557 (1), 169.0147 (11)
12.67	Heptagalloyl-glycose	9.95	1243.1405	C_55_H_40_O_34(6.1)_	1091.0774 (3), 939.0699 (3), 787.0776 (2), 635.1180 (100), 483.1152 (11), 331.1001 (2), 169 (5)
13.36	Octagalloyl-glucose	10.01	1395.1515	C_62_H_44_O_38(5.5)_	1243.1151 (15), 939.0714 (6), 787.0876 (5), 635.0144 (100), 483.0512 (9), 169.0147 (11)
14.10	Nonagalloyl glucose	7.27	1547.1580	C_69_H_48_O_42(2.1)_	1395.0005 (12), 1243.0834 (6), 1091.0251 (5), 787.0181 (100), 635.0872 (32), 331.0568 (3), 169.0144 (15)
15.04	Decagalloyl glucose	2.46	1699.9760	C_76_H_52_O_46(−9/8)_	1395.0517 (13), 1243.0596 (11), 1091.0118 (12), 939.0222 (12), 787.5201 (100), 635.0234 (17), 483.0348 (5), 169.0139 (33)
15.80	Nonadecagalloyl glucose	2.59	1851.1645	C_83_H_56_O_50(−6.5)_	1699.0443 (16), 1243.0735 (33), 1091.0737 (33), 939.0372 (100), 635.0240 (17), 483.0643 (15), 169.1235 (11)

**Table 2:** Phenolic compounds detected and identified in ethyl acetate fraction of *R. glabra* L. leaves by UHPLC-Q-orbitrap-HRMS.

R_t_, min	Identification	Content %	[M-H]^−^, *m*/*z*	Molecular Formula (Error, ppm)	MS/MS, *m*/*z* (Intensity, %)
0.524	Galloyl-quinic acid	4.62	343.0658	C_14_H_16_O_10(−3.6)_	191.0626 (12), 169.0158 (83), 153.0196 (100)
0.526	Digalloyl-quinic acid	0.12	495.0754	C_21_H_20_O_14(−6.2)_	313.2849 (17), 137.1587 (83), 169.1419 (30), 125.1001 (100)
4.84	Gallic acid	2.78	169.0141	C_7_H_6_O_5(−3.9)_	125.1237 (100)
5.19	Digalloyl glucose	3.74	483.0792	C_20_H_20_O_14(1.3)_	343.067 (80) 215.0462 (40), 197.0521 (90), 169.0144 (100)
5.87	Taxifolin hexoside	0.30	465.1014	C_21_H_22_O_12(−6.3)_	447.2103 (40), 303.2207 (40), 285.2176 (100), 259.2315 (60)
6.08	Trigalloyl-glucose	8.99	635.0875	C_27_H_24_O_18(−3.16)_	483.0756 (30), 321.0677 (100), 169.0128 (36)
6.70	Tetragalloyl-glucose	9.60	787.1052	C_34_H_28_O_22(6.0)_	635.0872 (15), 617.0122 (50), 485.0520 (25), 169.0155 (100)
6.82	Trigalloyl-levoglucosan	1.24	617.0795	C_27_H_22_O_17(0.9)_	465.0710 (30), 169.0150 (100), 125.0185 (70)
6.86	Rhaponticin	0.32	419.1335	C_21_H_24_O_9(−2.9)_	258.0085 (25), 197.0183 (100)
7.05	Quercetin	7.20	301.0345	C_15_H_10_O_7(−2.9)_	285.0060 (30), 257.0312 (40), 201.0034 (70), 153.0512 (100), 137.0700 (25)
7.05	Protocatechuic acid	0.64	153.0186	C_7_H_6_O_4(−2.9)_	109.0974 (100)
7.08	Valoneic acid dilactone	1.55	469.0062	C_21_H_10_O_13(2.8)_	469.0308 (30), 301.0716 (30), 169.0144 (100)
7.24	Penta-galloyl-glucose	9.62	939.1074	C_41_H_32_O_26(−3.7)_	787.1002 (100), 635.0173 (15), 483.0440 (20), 169.0136 (15)
7.26	1,5-di-O-galloyl-3,4- (S)-hexahydroxydiphenoyl protoquercitol	0.55	771.1062	C_34_H_28_O_21(1.5)_	301.0734 (30), 169.0459 (100), 164.0120 (15)
7.30	Hexagalloyl-glucoside	6.18	1091.1185	C_48_H_36_O_30 (−3.0)_	1091.0021 (20), 939.0980 (100), 787.0765 (40), 483.0462 (15), 183.0990 (20)
7.44	5-O-caffeoylshikimic acid (5-CSA)	0.50	335.0766	C_16_H_16_O_8(−1.9)_	179.0570 (50), 161.03 (50), 135.1540 (100)
7.44	Heptagalloylglucose	7.82	1243.1274	C_55_H_40_O_34(−4.3)_	1091.0145 (100), 939.0648 (15), 545.0664 (50), 335.0289 (20)
7.44	Isorhamnetin 7-O-[3-hydroxy-3-methylglutaroyl]hexoside	1.31	621.1198	C_31_H_26_O_14(−8.3)_	621.0642 (3), 559.067 (30), 519.0142 (51), 477.0470 (100)
7.52	Octagalloyl glucose	4.85	1395.1425	C_62_H_44_O_38(−0.9)_	1243.0308 (30), 1091.0384 (100), 939.0105 (15), 335.0473 (20)
7.75	Nonagalloyl glucose	3.18	1547.1521	C_69_H_48_O_42(−1.7)_	939.0597 (10), 787.0044 (100), 483.0369 (12), 169.0156 (15)
7.87	Caffeoylshikimic acid	2.23	335.0758	C_16_H_16_O_8(−4.3)_	179.0832 (50), 161.0273 (50), 135.1036 (100)
8.02	Hinokiflavone	0.22	537.0784	C_30_H_18_O_10(−8.0)_	537.0145 (15), 469.0572 (7), 271.0036 (20), 169.0142 (100), 125.0754 (30)
8.21	Myricetin glucoside	2.58	479.0796	C_21_H_20_O_13(−7.3)_	317.0141 (60), 316.0471 (100)
8.45	Quercetin-glucoside	4.78	463.0857	C_21_H_20_O_12(−5.3)_	301.065 (100), 299.0204 (30)
8.47	Resveratrol dimer-O-hexoside	1.69	615.1838	C_34_H_32_O_11(−5.5)_	453.0570 (100)
9.01	Luteolin hexoside	2.60	447	C_21_H_20_O_11(−1.9)_	301.053 (30), 285.0752 (100), 183.0180 (15)
9.26	Quercetin-rhamnoside	4.54	447.0922	C_21_H_20_O_11(−2.4)_	431.0137 (15), 301.0508 (100), 287.0170 (50), 160.0187 (5)
9.30	1,3-O-Caffeoyl-dihydrocaffeoylglycerol	1.20	416.1457	C_22_H_25_O_8(−4.7)_	255.0582 (100), 254.0537 (45), 253.0153 (40), 163.0917 (8), 161.0350 (20)
9.96	Apigenin-glucoside	1.74	431.1013	C_21_H_20_O_10__(__−6.7)_	343 (20), 271.068 (100), 217.0509 (25), 163.0370 (50)
11.42	Kaempferol	2.22	285.0423	C_15_H_10_O_6(6.4)_	255.0437 (100), 213.0350 (30), 165.0320 (5)

**Table 3:** DPPH scavenging activity expressed as IC_50_ values. Statistical significance was calculated vs. Trolox activity.

Tested Substances	IC_50_ [µg/mL] ± SD
*P. vera* ethyl acetate extract	21.45 ± 4.96 *
*R. glabra* ethyl acetate extract	13.77 ± 1.6 ***
Ascorbic acid	20.64 ± 3.46 ***
Trolox	33.6 ± 6.4

(*n* = 7, * *p* = 0.033, *** *p* < 0.001).

**Table 4:** MIC and MBC values for ethyl acetate plant extracts on selected Gram-positive and Gram-negative bacteria strains.

Microorganism		Extract of *P. vera* L.		Extract of *R. glabra* L.		Oxacillin	
		MIC [μg/mL]	MBC [μg/mL]	MIC [μg/mL]	MBC [μg/mL]	MIC [μg/mL]	MBC [μg/mL]
Gram-positive	*S. aureus* ATCC 700699 (MRSA)	125	500	62.5	250	1000	>2000
	*S. aureus* 8325-4 (MSSA)	7.8125	125	3.90625	62.5	1.953	3.906
	*S. epiderimidis* ATCC 51625 (MRSE)	125	500	62.5	250	1000	>2000
	*S. epiderimidis* ATCC 14990 (MSSE)	3.90625	62.5	1.953	31.25	1.953	3.906
	*S. mutans* ATCC 25175	62.5	250	31.25	125	62.5	250
	*B. cereus* ATCC 13061	62.5	125	31.25	125	62.5	250
Gram-negative	*E. coli* ATCC 35218	250	1000	125	1000	2000	>2000
	*P. aeruginosa* ATCC BAA-1744	500	>2000	250	2000	2000	>2000

## Data Availability

The data presented in this study are available on request from the corresponding author.
